# L-Carnitine Attenuates Testicular Dysfunction in Type 1 Diabetes Mellitus Via Modulation of Oxidative Stress, Inflammation, and miRNA Expression

**DOI:** 10.1007/s10753-025-02415-0

**Published:** 2025-12-18

**Authors:** Hero Khalid Mustafa, Khder Hussein Rasul, Azheen Subhi Abdulrahman, Harem Khdir Awla, Sana Moshari, Kamal A. Khidir

**Affiliations:** 1 Hawler Medical University, Erbil, Iraq; 2 Salahaddin University-Erbil, Erbil, Iraq; 3 Tishk International University, Erbil, Iraq; 4 Cihan University- Erbil, Erbil, Iraq; 5 RASTA Special Research Institute (RSRI), West Azerbaijan Science and Technology Park (WASTP), Urmia, Iran; 6 University of Leicester, Leicester, United Kingdom; 7 University of Raparin, Qeladize, Iraq

**Keywords:** L-carnitine, MiRNA, Oxidative stress, Pro-inflammatory cytokines, T1DM

## Abstract

Type 1 diabetes mellitus (T1DM) is a significant global health concern, adversely affecting various metabolic pathways and organ systems, including male reproductive health. This study aimed to investigate the protective effects of L-carnitine on T1DM-induced testicular dysfunction through its antioxidant and anti-inflammatory mechanisms. Male rats were divided into control, T1DM, L-carnitine, and T1DM + L-carnitine groups. After 8 weeks of treatment, serum glucose, blood HbA1c, testicular oxidative stress markers (TAC, TOS, MDA, and protein peroxidation), Pro-Inflammatory cytokines (IL-1β, IL-6, and TNF-α) and testosterone levels were measured. Additionally, the histological changes, sperm count, and miRNA expression (miR-155, miR-155-5p, and miR-132) were also studied. Results showed that T1DM significantly elevated oxidative stress markers, pro-inflammatory cytokines, and miRNA expression while reducing TAC, testosterone, sperm count, and Johnson’s score. Severe histological alterations, including germinal cell dissociation and atrophied seminiferous tubules, were observed in T1DM rats. L-carnitine treatment reduced oxidative stress and pro-inflammatory cytokines level, decreased lipid and protein peroxidation (> 3-fold), downregulated the miR-155, miR-155-5p, and miR-132 expression in diabetic rats (> 3-fold). Moreover, TAC (> 3-fold), testosterone levels (1-fold), sperm count, and testicular histological changes were restored by L-carnitine in diabetic rats. These findings suggest that L-carnitine ameliorates T1DM-induced testicular dysfunction through its dual antioxidant and anti-inflammatory properties. In line, L-carnitine significantly restored antioxidant capacity by directly scavenging reactive oxygen species, thereby reducing oxidative damage to lipids and proteins. These findings suggest that L-carnitine ameliorates T1DM-induced testicular dysfunction by enhancing antioxidant capacity and downregulating pro-inflammatory miRNAs (miR-155, miR-155-5p, miR-132).

## Introduction

Type 1 diabetes mellitus (T1DM) is an autoimmune disorder, which is characterized by the destruction of pancreatic beta cells, accounts for approximately 5–10% of all diabetes subjects [1]. T1DM is typically diagnosed early in life, which raises concerns about its long-term impact on reproductive health, as early-onset metabolic disturbances may coincide with critical periods of testicular development and maturation. Among these complications, its impact on male reproductive health is increasingly gaining attention, as it may contribute to infertility and reduced reproductive potential in men [2]. The adverse effects of T1DM on male reproductive health are multifaceted, involving structural and functional impairments of the testes and alterations in sperm parameters [3]. Animal studies have demonstrated that type 1 diabetes mellitus (T1DM) can lead to testicular atrophy, disruption of seminiferous tubule architecture, and impaired spermatogenesis through multiple mechanisms, including oxidative stress, apoptosis, endocrine dysregulation, autoimmune responses, and epigenetic alterations [4, 5].

Hyperglycemia associated with T1DM leads to excessive production of reactive oxygen species (ROS) and a concomitant depletion of antioxidant defenses, resulting in oxidative damage to testicular tissues [6]. Increased levels of oxidative biomarkers such as malondialdehyde (MDA) and protein carbonyl groups have been reported in testicular tissue under hyperglycemic condition [7, 8]. These changes can disrupt the integrity of sperm DNA, compromise membrane fluidity, and impair overall sperm functionality, further exacerbating infertility risks [9, 10]. In addition, diabetes induces a pro-inflammatory state that contributes to testicular dysfunction. In line with this issue, the elevated levels of pro-inflammatory cytokines, including interleukin-1 beta (IL-1β), interleukin-6 (IL-6), and tumor necrosis factor-alpha (TNF-α), have been observed in diabetic models [11]. These inflammatory mediators can impair Leydig cell function, reduce testosterone synthesis [12], and disrupt the blood-testis barrier [13], further impairing spermatogenesis.

Furthermore, emerging evidence indicates that microRNAs (miRNAs) play a critical role in mediating the adverse effects of diabetes on male reproductive function. Notably, miR-155 and miR-132 are among the dysregulated miRNAs implicated in key pathogenic pathways, including the NF-κB signaling pathway, oxidative stress responses, and mitochondrial-mediated apoptosis, all of which contribute to testicular dysfunction and impaired spermatogenesis [14, 15]. Despite these associations, the detailed molecular mechanisms by which miR-155 and miR-132 mediate testicular damage under diabetic conditions remain to be fully elucidated, justifying their focused investigation in this study.

L-carnitine is recognized for its potent antioxidant and anti-inflammatory properties [16]. It acts as a free radical scavenger, reducing oxidative stress by neutralizing ROS and enhancing the activity of endogenous antioxidant enzymes such as superoxide dismutase and glutathione peroxidase [17, 18]. Additionally, L-carnitine has been shown to reduce lipid peroxidation and protect cellular membranes against oxidative damage [19]. Its anti-inflammatory effects are mediated through the downregulation of pro-inflammatory cytokines, including IL-1β, IL-6, and TNF-α, thereby attenuating inflammation-induced tissue damage [20]. Given these properties, L-carnitine has emerged as a promising therapeutic agent for conditions associated with oxidative stress and inflammation, including diabetes-induced complications affecting the male reproductive system.

We specifically hypothesize that L-carnitine exerts its protective effects against T1DM-induced testicular dysfunction by attenuating oxidative stress and inflammation and by regulating key miRNAs implicated in testicular pathology. The present study aims to investigate the protective effects of L-carnitine against T1DM-induced testicular damage and reproductive dysfunction. Specifically, this research will examine key parameters such as oxidative stress biomarkers, pro-inflammatory cytokines, sperm quantity, testosterone concentration, and miRNA expression to elucidate the underlying mechanisms through which L-carnitine may ameliorate the hyperglycemia-related complications in the male reproductive system. By exploring the crosstalk between miRNA expression and the biochemical and histological changes in testicular tissue, this study seeks to provide novel insights into potential therapeutic strategies for preserving fertility and improving reproductive outcomes in diabetic individuals. The investigation will also highlight how alterations in miRNA expression influence the broader network of physiological processes involved in reproductive dysfunction under experimental hyperglycemic conditions.

## Materials and Methods

### Animals and Experimental Design

Male Wistar rats aged 8–10 weeks and weighing 200–250 g were housed in standard laboratory conditions with a temperature of 22 ± 2 °C, a 12-hour light/dark cycle, and unrestricted access to food and water. Prior to the study, animals were acclimated for one week to minimize stress. All experimental procedures were approved by the Institutional Animal Care and Use Committee (IACUC) of RASTA Special Research Institute (RSRI) (Approval Number: RSRI/UU/1325–2024), and the study adhered to the guidelines established by the National Institutes of Health for the care and use of laboratory animals.

The rats were randomly divided into four groups, each comprising eight animals: Control group: No interventions were applied, and the animals received standard laboratory chow and water. L-carnitine-only Group: Rats received oral L-carnitine (100 mg/kg/day) via gavage for 8 weeks [21]. The L-carnitine dose was selected based on prior research (Samir et al., 2018) that demonstrated its optimal efficacy in reducing oxidative stress in rodent models, while maintaining a favorable safety profile. This dose has been widely used to achieve therapeutic effects without inducing toxicity. T1DM Group: Type 1 diabetes mellitus (T1DM) was induced by a single intraperitoneal injection of streptozotocin (STZ, 55 mg/kg) dissolved in freshly prepared 0.1 M citrate buffer (pH 4.5). Diabetes was confirmed 72 h post-injection by measuring fasting blood glucose levels using a glucometer (Accu-Chek; Roche Diagnostics, Germany), with levels > 250 mg/dL considered diabetic. T1DM + L-carnitine Group: Diabetic rats were treated with oral L-carnitine (100 mg/kg/day) via gavage for 8 weeks. To simulate the therapeutic effect of L-carnitine, it was administrated one week after diabetes induction.

### Measurement of Serum Glucose and Blood HbA1c Levels

After 8 weeks of treatment, blood samples were collected via cardiac puncture under anesthesia induced by ketamine and xylazine cocktail (VOLZA, Netherlands). Serum glucose levels were measured using an enzymatic glucose oxidase assay kit (MyBioSource, Cat N: MBS7254179, USA). Blood HbA1c levels were determined using a commercial ELISA kit (MyBioSource, Cat N: MBS2701463, USA), following the manufacturer’s protocol. For ELISA assays, a minimum of six biological replicates per group were used. Each sample was measured in technical duplicate to ensure accuracy and reproducibility of the results.

### Testicular Oxidative Stress Markers

Testicular tissues were harvested immediately after euthanasia and rinsed in ice-cold phosphate-buffered saline (PBS). Homogenates were prepared by grinding the tissues in PBS (pH 7.4) at a ratio of 1 g tissue to 9 mL PBS. The homogenates were centrifuged at 10,000 × g for 10 min at 4 °C, and the supernatants were collected for biochemical assays. Total antioxidant capacity (TAC) level was measured using a colorimetric assay kit (Naxifer, Cat N: NS-15013, Iran). Total oxidative stress (TOS) was assessed using a commercial kit (Natos, CAT N: 15016, Iran). Malondialdehyde (MDA) as lipid peroxidation marker was quantified using the thiobarbituric acid-reactive substances (TBARS) method. Carbonyl groups (CG) as protein oxidation marker was measured using a commercial assay kit (MyBioSource, Cat N: MBS3806740, USA).

### Assessment of Pro-Inflammatory Cytokines

Testicular cytokine levels were determined from the same homogenates prepared for oxidative stress markers. Testicular levels of IL-1β (Elabsciences, Cat N: E-EL-H0149, USA), IL-6 (Elabsciences, Cat N: E-EL-R0015, USA), and TNF-α (Elabsciences, Cat N: E-EL-M3063, USA) were quantified using specific commercial ELISA kits according to the manufacturer’s instructions.

### Measurement of Serum Testosterone Levels

Serum testosterone concentrations were measured using a competitive enzyme-linked immunosorbent assay (ELISA) kit (Elabsciences, Cat N: E-EL-0155, USA) according to the manufacturer’s instructions.

### Histological Analysis and Johnson’s Score

Testicular tissues were fixed in 10% neutral buffered formalin for at least 24 h and processed for paraffin embedding. Section (5 μm) were cut by rotary microtome (LKB, UK) and stained with hematoxylin and eosin (H&E) [22, 23]. Histological examination was performed under a light microscope (Leica, Germany). All histopathological evaluations were conducted by a single experienced and blinded histopathologist to ensure scoring consistency; therefore, interobserver variability was not assessed in this study. Spermatogenesis was evaluated using Johnson’s scoring system, which grades germ cell development on a scale of 1 to 10, with 10 indicating normal spermatogenesis [24].

### Sperm Count Analysis

Epididymal sperm count was determined by excising the cauda epididymis and mincing it in 2 mL of pre-warmed PBS (37 °C). The suspension was incubated for 10 min to release sperm, filtered through gauze to remove debris, and sperm count was determined using a hemocytometer under a light microscope [25].

### Gene Expression Analysis of miR-155, miR-155-5p, and miR-132

Total RNA was extracted from testicular tissue using TRIzol reagent (YTzol Pure RNA Yekta Tajhiz, Cat N: A20211, Iran) following the manufacturer’s protocol. RNA concentration and purity were determined using a NanoDrop spectrophotometer (MASTERO, Tiwan). Complementary DNA (cDNA) synthesis was performed using a miRNA-specific reverse transcription kit (Sinaclon, Iran). Quantitative real-time PCR (qRT-PCR) was conducted using SYBR Green Master Mix (Parstous, Cat N: 793295, Iran) on a Pantamer real-time PCR system (Pantamer, Germany). As shown in Table 1, primer sequences were designed for miR-155, miR-155-5p, miR-132, and U6 small nuclear RNA (internal control). The expression levels of target microRNAs were normalized to U6 small nuclear RNA, which served as a stable internal reference due to its consistent expression across experimental groups. Relative expression levels were calculated using the 2^−ΔΔCt^ method. For qPCR assays, a minimum of six biological replicates per group were used. Each sample was measured in technical triplicate to ensure accuracy and reproducibility of the results.

**Table 1:** The primer sequences of MiRNAs and housekeeping miRNA

Primers miRNA	Forward	Reverse	AT
miR-155	5’-CGACTGAGGTTTGTGAGCTGGT-3’	5’-GTGCAGGGTCCGAGGT-3’	58 °C
miR-155-5p	5’-ACGACTGAGGTTTGTGAGCTGGT-3’	5’-GTGCAGGGTCCGAGGT-3’	59 °C
miR-132	5’-CGACCATGGCTGTAGACTGTTAA-3’	5’-GTGCAGGGTCCGAGGT-3’	60 °C
RNU6	5’-CTCGCTTCGGCAGCACA-3’	5’-AACGCTTCACGAATTTGCGT-3’	60 °C

### Statistical Analysis

All data were analyzed using GraphPad Prism version 9 (GraphPad Software, USA). Results were expressed as mean ± standard deviation of the mean (STD). Group comparisons were made using one-way analysis of variance (ANOVA) followed by Tukey’s post hoc test. A p-value < 0.05 was considered statistically significant.

## Results

### Serum Glucose and Blood HbA1c Levels

**Fig. 1:**
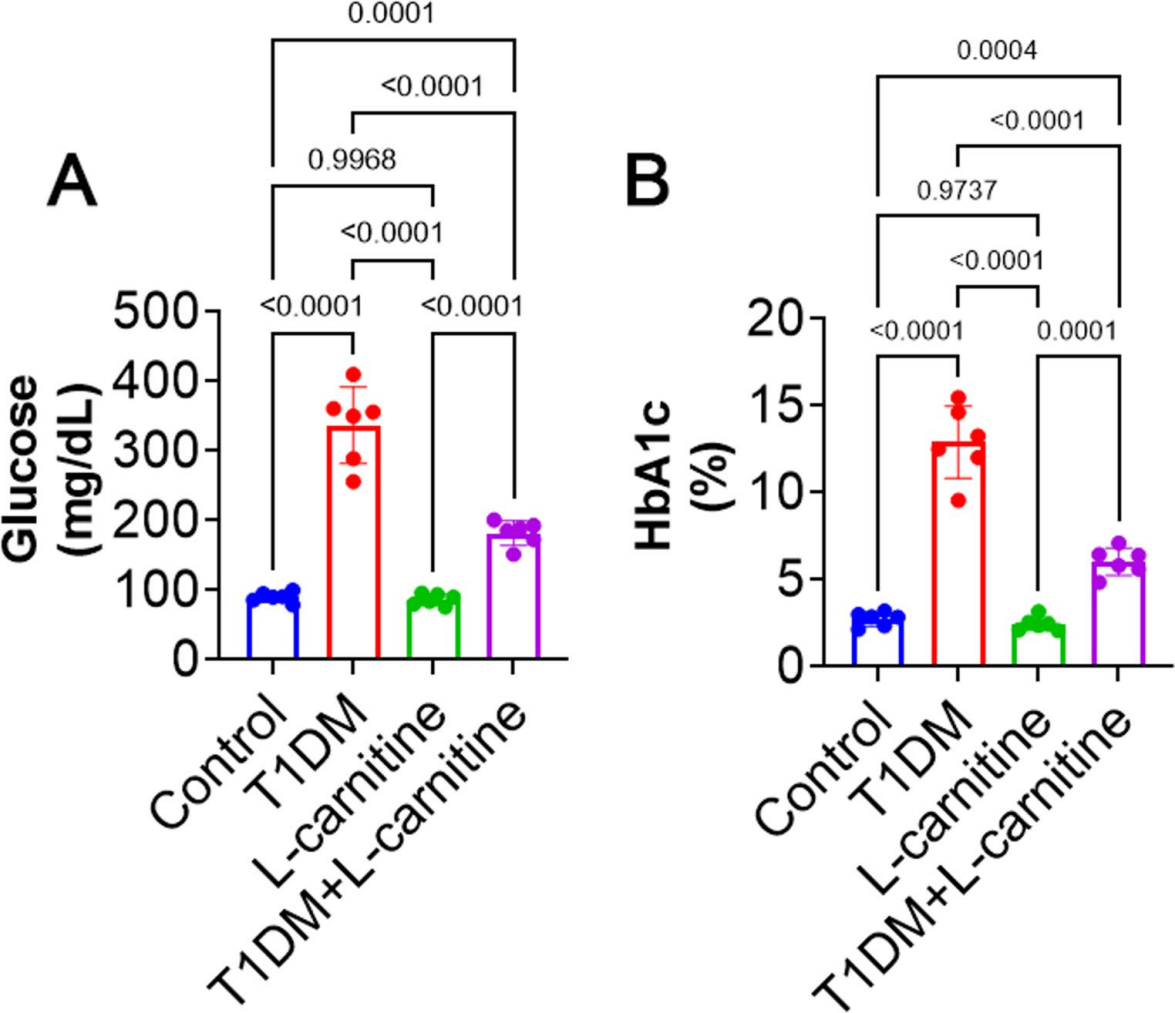
The level of (**A**) serum glucose and (**B**) blood HbA1c in different groups. All data are presented in Mean ± SD (*n* = 6 rat/group)

At the end of the current research, STZ induced T1DM rats still have high glucose and HbA1c levels as indicator of diabetes compared to the control group. No significant difference (*p* > 0.05) was observed in L-carnitine-only and control group rats. However, L-carnitine treatment in the T1DM group (T1DM + L-carnitine) resulted in a significant reduction (*p* < 0.0001) in serum glucose levels and blood HbA1c at the end of the study compared to T1DM rats (Fig. 1A and B).

### Testicular TAC, TOS, Lipid Peroxidation, and Protein Peroxidation

**Fig. 2:**
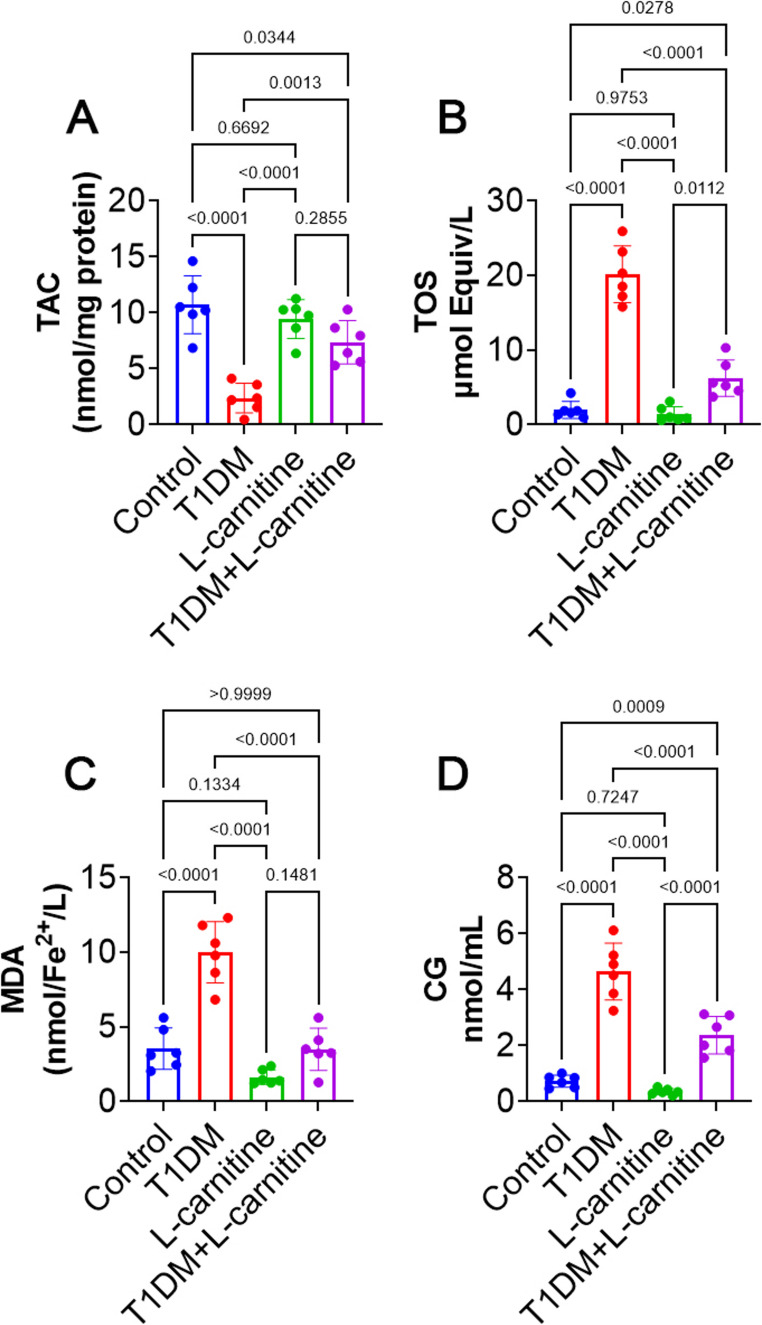
Effect of experimental-induced type 1 diabetes mellitus (T1DM) and protective role of L-carnitine administration on testicular (**A**) total antioxidant capacity (TAC), (**B**) total oxidant status (TOS), (**C**) malondialdehyde (MDA), and (**D**) protein peroxidation marked with carbonyl group (CG) levels in different groups. All data are presented in Mean ± SD (*n* = 6 rat/group)

The protective effect of L-carnitine against T1DM-induced oxidative stress was assessed by evaluating testicular TAC and TOS levels. Diabetes led to significantly reduce (*p* < 0.0001) and increase (*p* < 0.0001) of TAC and TOS respectively in the T1DM group compared to the control group, with no notable changes (*p* > 0.05) observed between L-carnitine-only group and the control group rats. However, L-carnitine-treated T1DM rats exhibited significantly higher TAC levels (*p* < 0.01) and significantly lower TOS level (*p* < 0.0001) than T1DM rats (Fig. 2A and B).

In addition, testicular lipid and protein peroxidation were analyzed by measuring MDA and CG levels respectively. In T1DM rats, MDA and CG levels were significantly elevated (*p* < 0.0001) compared to the control group. No significant difference (*p* > 0.05) was noted between the control and L-carnitine-only groups. Nonetheless, L-carnitine treatment significantly reduced (*p* < 0.0001) MDA and CG levels in T1DM + L-carnitine group rats compared to T1DM group rats (Fig. 2C and D).

### Pro-Inflammatory Cytokine Levels

**Fig. 3:**
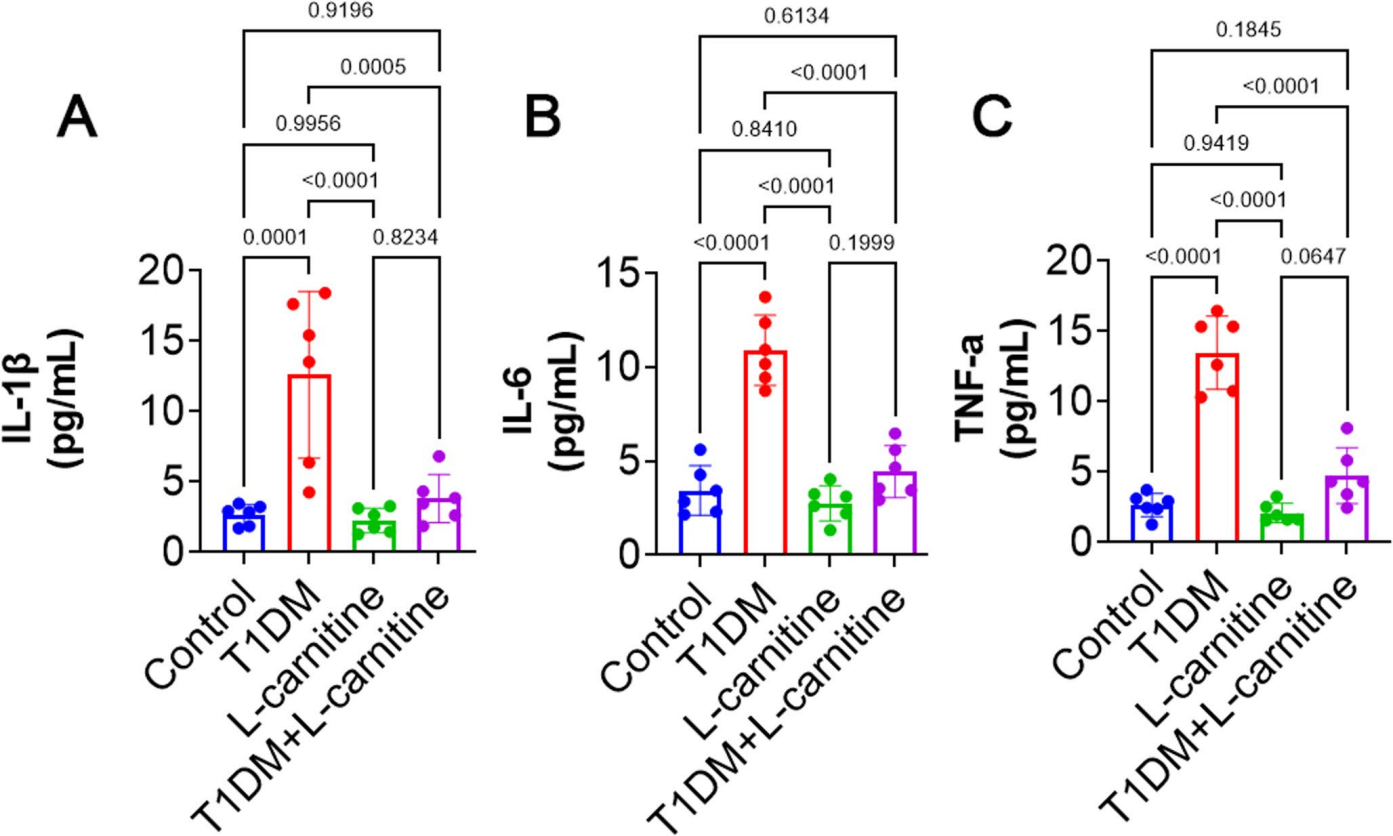
Effect of experimental-induced type 1 diabetes mellitus (T1DM) and protective role L-carnitine administration on testicular levels of (**A**) interlukine-1β (IL-1β), (**B**) interlukine-6 (IL-6), and (**C**) tumor necrosis factor-α (TNF-α) in different groups. All data are presented in Mean ± SD (*n* = 6 rat/group)

Testicular levels of IL-1β, IL-6, and TNF-α were significantly increased in the T1DM group compared to controls. No significant changes (*p* > 0.05) were observed between the control and L-carnitine-only groups. However, L-carnitine-treated T1DM rats exhibited significant reductions in testicular IL-1β (*p* = 0.0005), IL-6 (*p* < 0.0001), and TNF-α (*p* < 0.0001) levels compared toT1DM animals (Fig. 3A, B and C).

### Serum Testosterone Levels

**Fig. 4:**
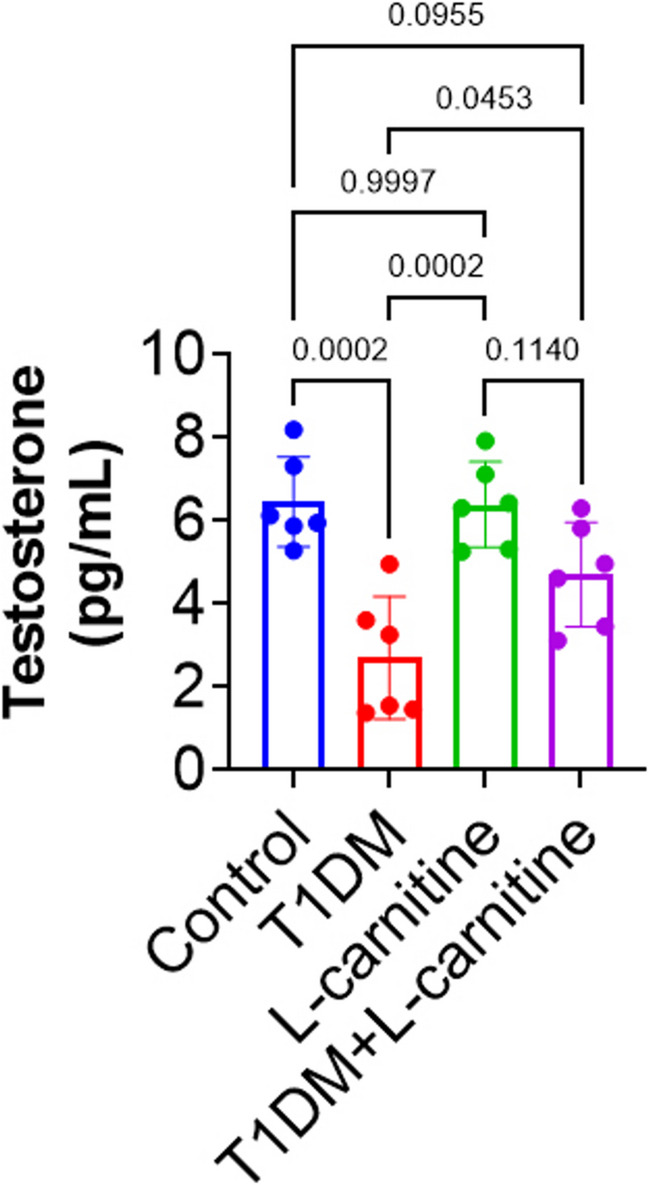
Effect of experimental-induced type 1 diabetes mellitus (T1DM) and protective role L-carnitine administration on serum level of testosterone in different groups. All data are presented in Mean ± SD (*n* = 6 rat/group)

Serum testosterone levels were significantly reduced (*p* < 0.00001) in the T1DM group compared to control group rats. No significant changes were detected between rats of control and L-carnitine-only groups. But, L-carnitine-treated T1DM rats displayed significantly higher testosterone levels (*p* < 0.05) than T1DM rats (Fig. 4).

### Histological Findings and Sperm Count

**Fig. 5:**
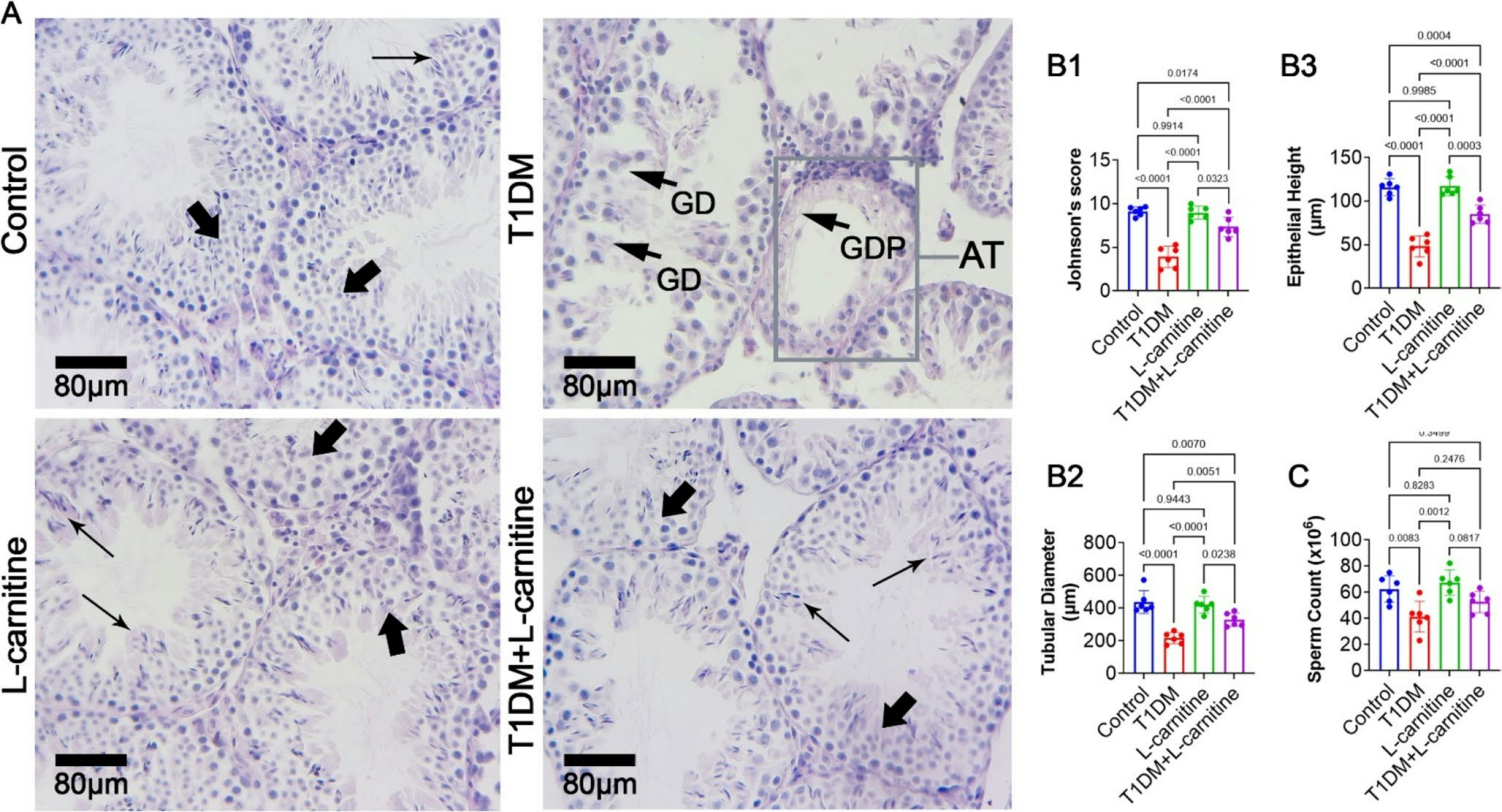
Effect of experimental-induced type 1 diabetes mellitus (T1DM) and protective role L-carnitine administration on histological characteristic of testicles and sperm count. (**A**) cross-sections of the testicular tissue. Normal seminiferous tubules with intact spermatogenesis (thick arrows) and spermiogenesis (thin arrows) noted in control and L-carnitine groups. The section from T1DM group represented severe germinal cells dissociation (GD), germinal cells depletion (GDP) and atrophied seminiferous tubule (AT). In contrast, L-carnitine has ameliorated the T1DM-induced damages marked by tubules with improved spermatogenesis and spermiogenesis (H&E staining), (**B1-3**) histomorphomteric parameters of the cross-sections, and (**C**) mean changes in the total sperm count in different groups. All data are presented in Mean ± SD (*n* = 6 rat/group)

Histological section from the T1DM group revealed edema in the interstitial connective tissue, severe germinal cell dissociation, depletion, and atrophied tubules. These abnormalities were ameliorated in the L-carnitine-treated T1DM group (Fig. 5A). In addition, to evaluate histological changes, Johnson’s score was used. The T1DM group showed a significant reduction (*p* < 0.0001) in Johnson’s score compared to controls. No significant differences were observed between the control and L-carnitine-only groups. L-carnitine-treated T1DM rats displayed significant improvement (*p* < 0.0001) in Johnson’s score compared to T1DM rats (Fig. 5B1). Moreover, tubular diameter (Fig. 5B2) and epithelial height (Fig. 5B3) were reduced significantly (*p* < 0.0001) in T1DM rats compared to rats of control group. No significant changes (*p* > 0.05) were found between the control and L-carnitine-only groups. The tubular diameter and epithelial height increased significantly in L-carnitine T1DM treated rats compared to T1DM rats. Furthermore, the T1DM group rats demonstrated a significant reduction (*p* = 0.008) in sperm count compared to controls. No significant changes (*p* > 0.05) were found between the control and L-carnitine-only groups. The sperm count increased but it was not statistically significant in L-carnitine T1DM treated rats compared to T1DM animals (Fig. 5C).

### *miR-155*,* miR-155-5p and miR-132 Expression*

**Fig. 6:**
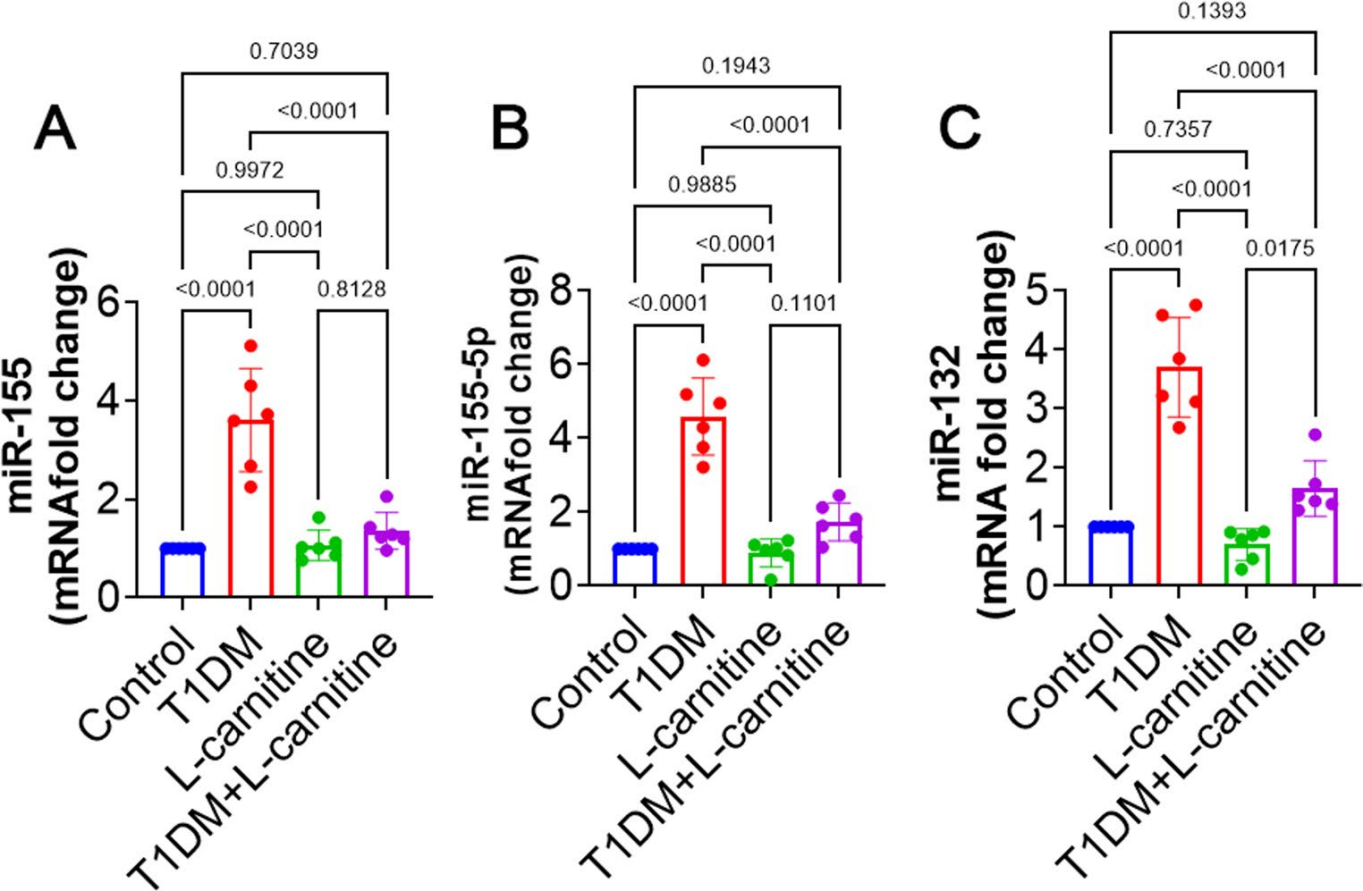
Effect of experimental-induced type 1 diabetes mellitus (T1DM) and protective role L-carnitine administration on (**A**) miR-155, (**B**) miR 155-5p, and (**C**) miR-132 expression in different groups. All data are presented in Mean ± SD (*n* = 6 rat/group)

The T1DM group rats exhibited a significant increase (*p* < 0.0001) in miR-155, miR-155-5p and miR-132 expression compared to the control group. In terms of evaluated miRNAs of current research, no significant differences (*p* > 0.05) were found between L-carnitine-only and control groups. However, L-carnitine-treated T1DM rats revealed significantly lower (> 3-fold change, *p* < 0.0001) expression of mentioned miRNAs than T1DM rats (Fig. 6).

## Discussion

The present study highlighted the detrimental effects of T1DM on the male reproductive system, focusing on the spermatogenesis and spermiogenesis development (marked with Johnson’s score), alterations in sperm count, and testicular testosterone imbalances. Additionally, the research explored the potential protective effects of L-carnitine, an antioxidant and anti-inflammatory agent, in ameliorating the impairments caused by T1DM-induced oxidative stress and pro-inflammatory cytokines level, two key factors involved in T1DM-related testicular dysfunction. It is established that T1DM significantly disrupts the antioxidant/oxidant balance in testicular tissue, leading to severe lipid and protein peroxidation in both germinal and somatic cells, thereby inducing oxidative stress [7, 26]. This is consistent with a study that reported an increase of oxidative biomarkers, such as MDA and protein carbonyl groups, in the testes of diabetic animals [8]. These are supporting results of the current study in which rats with T1DM exhibited high levels of MDA and protein peroxidation, along with a marked reduction in TAC and an increase in TOS. Notably, the administration of L-carnitine ameliorates these alterations, as evidenced by reduced MDA and protein carbonyl levels, increased TAC, and decreased TOS content. These results underscore the potential of L-carnitine as a therapeutic agent for ameliorating T1DM-induced oxidative damage at both lipid and protein levels.

Inflammation is another critical factor contributing to testicular dysfunction in T1DM. Elevated levels of pro-inflammatory cytokines have been consistently reported in diabetic animal models [11]. Inflammatory mediators not only damage testicular architecture by impairing the blood-testis barrier [13] but also disrupt the function of Leydig cells, which are responsible for testosterone synthesis [12, 27]. Furthermore, the overproduction of pro-inflammatory cytokines in the interstitial connective tissue recruit’s immune cells, which, in turn, exacerbate oxidative stress within the testicular microenvironment [28, 29]. These are in align with the results of present study findings, as T1DM rats exhibited significantly elevated levels of IL-1β, IL-6, and TNF-α as pro-inflammatory cytokines. However, L-carnitine treatment resulted in a significant reduction in their levels, suggesting its role in modulating the inflammatory response. This anti-inflammatory effect of L-carnitine aligns with previous study reduced inflammation by downregulating cytokine production [20].

The role of testosterone in male reproductive health should not be overlooked. Testosterone is crucial for maintaining spermatogenesis and overall male reproductive function [30, 31]. In the present study, T1DM rats exhibited significantly lower testosterone levels, which aligns with previous research highlighting the negative impact of diabetes on testosterone synthesis [27]. However, L-carnitine treatment resulted in a significant increase in testosterone levels, supporting its role in improving hormonal balance and enhancing testicular function in diabetic animals. Based on these findings, L-carnitine may modulate pro-inflammatory cytokine production in the testicular microenvironment by preserving Leydig cell function, which in turn could support testosterone synthesis and release. However, while an increase in sperm count was observed in the L-carnitine-treated group, this change did not reach statistical significance and should therefore be interpreted with caution regarding therapeutic efficacy.

The results of this study offer valuable insights into the role of miRNAs, specifically miR-155, miR-155-5p, and miR-132, in the pathophysiology of testicular dysfunction in T1DM. Indeed, miR-155 has long been recognized as a key player in inflammatory responses, particularly in conditions of oxidative stress [32]. Elevated miR-155 expression has been associated with increased levels of pro-inflammatory cytokines, which contribute to the inflammatory milieu in diabetic tissues [33, 34]. In this study, results revealed significantly higher miR-155 expression simultaneously with severe increase in pro-inflammatory cytokines in the T1DM group, suggesting that miR-155 may be directly involved in the heightened inflammatory response seen in hyperglycemic testicular tissue. Furthermore, L-carnitine’s ability to reduce miR-155 expression in T1DM rats provides compelling evidence for its potential as an anti-inflammatory agent, which could contribute to the alleviation of oxidative stress and testicular damage observed in diabetes. The results for miR-155-5p, a variant of miR-155, closely mirrored those of miR-155, with significant upregulation observed in T1DM rats and a marked reduction following L-carnitine treatment. Regarding miR-132, it has been implicated in various pathological conditions, including inflammation and fibrosis, particularly in the context of hyperglycemia [35]. Previous studies have demonstrated that miR-132 regulates several critical cytokines, such as IL-1β, IL-6, TNF-α, and TGF-β, which are central to the inflammatory and fibrotic pathways exacerbating diabetic complications [36]. These findings are in supporting the results of current research which revealed a significant increase in miR-132 expression in the T1DM group, suggesting that miR-132 may contribute to the chronic inflammation and fibrosis commonly seen in diabetes-induced tissue damage. The ability of L-carnitine to reduce miR-132 expression in T1DM rats provides further evidence of its potential to modulate inflammatory responses and prevent fibrosis, which are critical in preserving testicular function in diabetes. Therefore, the regulatory effects of L-carnitine on miRNA expression suggest that it may act as a molecular modulator, exerting both antioxidant and anti-inflammatory effects. These findings suggest a potential role for L-carnitine as a therapeutic candidate in managing reproductive health issues associated with diabetes.

The histological findings in this study further support the notion that oxidative stress and inflammation contribute to testicular damage in T1DM. T1DM rats exhibited severe histological changes which is consistent with earlier studies demonstrating that antioxidants can ameliorate testicular histological alterations in diabetic animals [37]. The restoration of testicular integrity in L-carnitine-treated T1DM animals suggests that L-carnitine plays a role in maintaining the structural integrity of the testes, which is critical for sperm production and fertility. Moreover, in the present study, T1DM rats exhibited a significant reduction in sperm count, which is in line with previous reports on the adverse effects of diabetes on spermatogenesis [2, 3]. Although L-carnitine treatment resulted in an increase in sperm count in T1DM rats, this change did not reach statistical significance. This trend suggests that a longer treatment duration may be required for L-carnitine to effectively enhance spermatogenesis, potentially through the modulation of the antioxidant–oxidant balance and inflammatory pathways via regulation of miR-155, miR-155p, and miR-132 expression. The observed improvement in sperm count is encouraging and suggests that L-carnitine may hold potential as a therapeutic strategy to support fertility in diabetic men, although further studies are needed to confirm its efficacy.

## Conclusion

This study provides evidence of the detrimental impact of T1DM on male reproductive health, highlighting the important roles of oxidative stress, inflammation, and hormonal imbalances in testicular dysfunction. The findings suggest that L-carnitine may offer therapeutic benefits by attenuating these adverse effects through multiple mechanisms. L-carnitine appeared to enhance antioxidant capacity by scavenging reactive oxygen species and supporting endogenous antioxidant defenses, thereby reducing oxidative damage to lipids and proteins. Additionally, it was associated with reduced pro-inflammatory cytokine production through the downregulation of miR-155, miR-155-5p, and miR-132, key regulators of inflammatory pathways. This combined antioxidant and anti-inflammatory effect may contribute to partial recovery of testosterone synthesis. Furthermore, L-carnitine treatment was linked to improvements in testicular morphology and function, with a trend toward enhanced spermatogenesis, although not all parameters, such as sperm count, reached statistical significance. These results indicate that L-carnitine holds potential as an adjunct therapeutic approach to mitigate diabetes-associated male reproductive dysfunction. However, further studies are necessary to confirm these findings, clarify the extent of functional recovery, and elucidate the underlying mechanisms.

## Study Limitations

This study has several limitations that warrant consideration. First, the investigation employed a single dose of L-carnitine (100 mg/kg/day) without conducting dose-response analyses, which limits the understanding of the optimal therapeutic range and potential dose-dependent effects. Second, the absence of a diabetic group treated with insulin precludes direct comparison of L-carnitine’s efficacy with standard diabetes management, which could provide valuable clinical context. Third, the relatively short experimental duration of eight weeks may not fully capture the chronic and progressive nature of type 1 diabetes mellitus and its long-term impact on testicular function. Finally, while biochemical and histological parameters were assessed, the study did not include functional fertility evaluations, such as mating trials or fertility indices, which are essential to confirm the reproductive recovery suggested by the molecular and cellular findings. Future studies addressing these limitations will help to more comprehensively evaluate L-carnitine’s therapeutic potential in diabetic reproductive dysfunction.

## Data Availability

All the data used and analyzed during the current study are available from the corresponding author on reasonable request.
